# Genetic marker anchoring by six-dimensional pools for development of a soybean physical map

**DOI:** 10.1186/1471-2164-9-28

**Published:** 2008-01-22

**Authors:** Xiaolei Wu, Guohua Zhong, Seth D Findley, Perry Cregan, Gary Stacey, Henry T Nguyen

**Affiliations:** 1 Division of Plant Sciences and National Center for Soybean Biotechnology, University of Missouri-Columbia, Columbia, MO 65211, USA; 2 Soybean Genomics and Improvement Laboratory, USDA-ARS, Beltsville, MD 20705, USA; 3 Department of Biochemistry; Department of Molecular Microbiology and Immunology, University of Missouri-Columbia, Columbia, MO 65211, USA

## Abstract

**Background:**

Integrated genetic and physical maps are extremely valuable for genomic studies and as important references for assembling whole genome shotgun sequences. Screening of a BAC library using molecular markers is an indispensable procedure for integration of both physical and genetic maps of a genome. Molecular markers provide anchor points for integration of genetic and physical maps and also validate BAC contigs assembled based solely on BAC fingerprints. We employed a six-dimensional BAC pooling strategy and an *in silico *approach to anchor molecular markers onto the soybean physical map.

**Results:**

A total of 1,470 markers (580 SSRs and 890 STSs) were anchored by PCR on a subset of a Williams 82 *Bst*Y I BAC library pooled into 208 pools in six dimensions. This resulted in 7,463 clones (~1× genome equivalent) associated with 1470 markers, of which the majority of clones (6,157, 82.5%) were anchored by one marker and 1106 (17.5%) individual clones contained two or more markers. This contributed to 1184 contigs having anchor points through this 6-D pool screening effort. In parallel, the 21,700 soybean Unigene set from NCBI was used to perform *in silico *mapping on 80,700 Williams 82 BAC end sequences (BES). This *in silico *analysis yielded 9,835 positive results anchored by 4152 unigenes that contributed to 1305 contigs and 1624 singletons. Among the 1305 contigs, 305 have not been previously anchored by PCR. Therefore, 1489 (78.8%) of 1893 contigs are anchored with molecular markers. These results are being integrated with BAC fingerprints to assemble the BAC contigs. Ultimately, these efforts will lead to an integrated physical and genetic map resource.

**Conclusion:**

We demonstrated that the six-dimensional soybean BAC pools can be efficiently used to anchor markers to soybean BACs despite the complexity of the soybean genome. In addition to anchoring markers, the 6-D pooling method was also effective for targeting BAC clones for investigating gene families and duplicated regions in the genome, as well as for extending physical map contigs.

## Background

The complete sequencing of the genomes of major crop plants, including soybean, promises to revolutionize the study of these plants, especially for crop improvement. Soybean was recommended as the model genome for the *Phaseoloid *legumes [1] because of its economic importance, moderate genome size, existing resources and genomic and genetic tools [2]. Soybean is also an excellent model for studying the evolutionary dynamics of a paleopolyploid genome [3]. A research strategy for advancing soybean genomics and its applications was defined by the community based on the availability of numerous genomic resources for soybean and other legumes [2].

Soybean is a paleopolyploid genome consisting of ~1100 Mbp [4], which is thought to be the product of two to three rounds of genome duplication, polyploidization and diploidization in the last 45 MY [5-7]. New evidence suggests that the most recent of these large-scale duplications may have occurred a mere 1 to 3 MY ago [3]; thus, some duplicated regions are highly similar at the sequence level, with 86–100% sequence identity [8]. Some duplicate regions have nearly identical fingerprint patterns that can be distinguished only at the sequence level [9]. The duplicated nature of the soybean genome presents a significant challenge for genomic and biological research to identify the key regions important for determining agronomic traits. The soybean community has come together to develop a 'gold standard' physical and genetic map resource to facilitate this research [3]. Integrated genetic and physical maps are extremely valuable for map-based gene isolation, comparative genome analysis, as sources of sequence-ready clones for genome sequencing projects and as important references for assembling of whole genome shotgun sequences. Such a whole genome shotgun approach is currently being employed by the Department of Energy-Joint Genome Institute to sequence the soybean genome [3].

The soybean composite genetic map is well developed consisting of 20 linkage groups with 1,849 markers, including 1,015 SSRs, 709 RFLPs, 73 RAPDs, 24 classical traits, six AFLPs, ten isozymes, and 12 others [10]. Another 1141 sequence-based genetic markers were mapped to the soybean genome map [11]. A physical map of the 'Forrest' genotype was initially developed from 78,001 BAC and BIBAC clones representing 9.6 haploid genomes [12]. A physical map of the Williams 82 genotype is also available [13,14] and was constructed by fingerprinting 67,968 BAC clones from a *Bst*Y I library and 40,320 clones from a *Hind *III library [14]. The current version of this map consists of 92,272 BAC clones in 1893 contigs with 30,000 singletons [14]. Recent improvements to this map include the addition of 222 mapped SSR markers that helped resolve map inconsistencies [14]. However, a number of contigs still exist in which anchored genetic markers come from different linkage groups. These inconsistencies are likely the result of the recent soybean genome duplication and represent homoeologous regions of the genome.

Screening of a BAC library is an indispensable procedure for integration of both physical and genetic maps of a genome and is usually performed with a hybridization-based approach, such as overgo probe hybridization, or a PCR-based approach. Overgo probes have gained in popularity as markers for large-scale physical mapping of both plant and animal genomes [15,16]. They are particularly useful for screening large-insert libraries to identify clones containing gene-specific STS (sequence tagged site) markers that can be used in contig assembly. However, overgo markers suffer from the limitations of hybridization and often cannot determine the locus-specific contig. This is especially problematic in a genome with recent duplications, such as soybean. In contrast, with appropriate primer design, PCR-based markers may be locus-specific, defined by single bands on agarose gels.

Theoretical analysis of library screening using a N-dimensional pooling strategy [17,18] led to a more efficient six-dimensional pooling strategy for PCR screening of BACs from large libraries of sorghum [19] and maize [20]. This pooling strategy not only uniquely defines individual clones and efficiently eliminates false positives, but also reduces the tedious task of individual clone verifications, by simultaneously comparing any three different configurations of positive pools after one PCR screening without the need for additional PCR screening, such as the superpool strategies used in human [21], bovine [22], grapevine [23], and sunflower [24]. In contrast to hybridization-based approaches (e.g., using overgoes), PCR-based screening of BAC pools also allows for rapid verification of results by sequencing of the resulting PCR product. This provides an accurate estimate of false positives and aids in the identification of paralogous regions that complicate the alignments between FPC contigs and genetic markers, since the size of the PCR products from paralogous regions may be identical.

*In silico *anchoring is an alternative strategy widely used in anchoring BAC clones to a genetic map [25], in the construction of comparative maps [26] and in mapping STS markers onto a sequence-based map [23,27,28]. The *in silico *mapping approach was demonstrated to be robust in rice using stringent cutoff parameters (≥95% identity over regions ≥40 bp in length) and filtering repetitive sequences [25].

In this study, our goal was to obtain at least 1500 connections between the soybean genetic and physical maps using a 6-D, PCR based strategy. Based on the distribution of the more than 1000 SSR markers [10] and additional 1100 gene-based markers on the genetic map [11], we selected 1500 genetic markers for our study. We also designed primers from suspected homoeologous regions and from BAC-end sequences (BES) to explore the utility of the 6-D pools for targeting duplicated contigs and assisting contig merging. We also mined the available BES and database sequences to anchor markers via *in silico *approaches. These methods resulted in the placement of thousands of molecular markers onto the 'Williams' 82 BAC contig physical map, establishing useful connections to the soybean genetic map. All these resource are available to the soybean scientific community [13].

## Results

### 6-D BAC pool generation

A *Bst*Y I BAC library was constructed for the soybean cultivar Williams 82 in vector pCUGIBAC1 (Christopher Saski and Jeff Tomkins, Clemson University Genomics Institute). The library consists of 92,160 clones stored in 240 384-well microtiter plates. To determine the size distribution of BAC clones in the library, the insert size of 196 randomly sampled clones was estimated after *Bst*Y I digestion. The average insert size is 150 kb, with a range of 70 to 300 kb (data not shown). More than 80% of the clones are larger than 100 kb. Given the average insert size and estimated size of the soybean genome, the library represents approximately 12.5 haploid equivalents. The library can be obtained through the Clemson University Genomics Institute.

**Figure 1:**
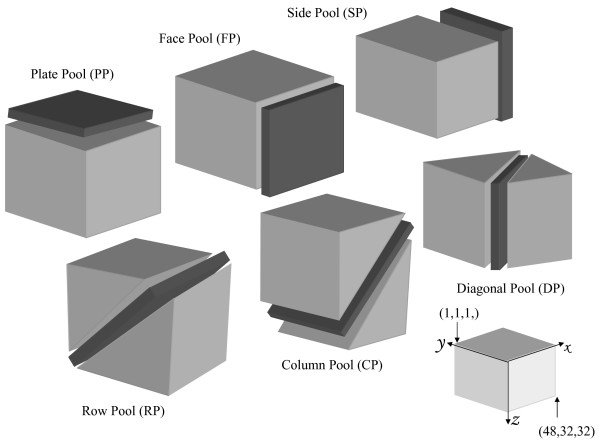
**Schematic display of six-dimensional BAC pooling strategy**. One hundred twenty eight 384-well microtiter plates containing 49,192 individual BAC clones were conceptually arranged in a cubic stack consisting 32 layers × 48 columns × 32 rows. The stack was pooled on the six unique coordinate axes as shown to generate a total of 208 DNA pools.

The 6D pooling design is illustrated in Figure 1. The pooling was performed using a modification of the established method used in sorghum [19] as dictated by the soybean genome size. We selected 128 384-well plates with the best uniform culture growth to generate BAC DNA pools. A total of 49,192 BAC clones were pooled in six distinct directions to generate 208 BAC pools. Each BAC pool consists of 32 sub-pools except the Side Pool (SP) with 48 sub-pools. The number of clones per pool is 1,536 clones or 1024 clones (one-fifth or one-seventh genome equivalent). As a reflection of the soybean genome size, these numbers are higher than those used for sorghum (1024 or 768 clones/pool) [19] but lower than those used for maize (2,304 clones/pool) [20]. Given the genome coverage, an average of 6.6 BAC clones should be identified for each locus probed. The six dimensional pools should have an expected 36~42 positives for each marker investigated.

### Anchoring genetic markers using the 6D pools

As a control, we constructed a BAC pool consisting of all of the 208 pools. This mixture was used to test all primer sets before probing the 6D pools and was also helpful in optimizing PCR conditions to improve detection. In this way, we were assured that positive clones were present in the pools before investing time and money for pool screening. A subset of pools (23) was then used for PCR pre-screening to select primers which produced a number of positives with a defined range for further screening. The primers with many positives (>10) or no positive in the 23 subset pools out of 208 pools were eliminated from further screening. In each step of screening, the Williams 82 genomic DNA was used as a control to easily identify which PCR amplicon corresponded to the targeted marker or gene. We observed in several cases that primers would generate multiple bands when using BAC DNA as the template, but only a single band was observed using genomic DNA as the PCR template.

The PCR screening workflow is illustrated in additional file 1. In this study, 1700 primer sets were tested against a subset of BAC pools (23 pools plus genomic DNA as control). We used 1504 primer pairs having 1~10 positives out of the 23 subset pools for full screening of the 208 pools. The failure of 196 primer sets was due to a variety of reasons; such as, PCR failure (76 primer pairs failed to amplify or generated weak bands on agarose gels), more than 10 positives out of the 23 subset pools (61 primer sets may have amplified duplicated regions producing PCR products of similar size that could not be distinguished on an agarose gel), lack of primer specificity (39 primer pairs amplified virtually every BAC pool and likely represent repetitive sequences in the genome), or representing apparently unclonable regions (20 primer pairs amplified a band from genomic DNA but did not amplify any of the BAC pools suggesting the lack of these regions in the *Bst*Y I library).

The six-dimensional pools were screened with 1,504 PCR-based markers, including 563 SSRs, 783 SNP-containing STSs and 158 STS markers without genetic location information. Eventually, 1470 clear PCR products from the Williams 82 genomic DNA amplified with 1320 primer sets were de-convoluted. Among 1320 anchored markers, 1200 primer pairs (90.9%) amplified a unique band and 120 primer pairs (8.1%) amplified multiple distinct bands (2~4 bands/primer set, total 270 bands). We assumed each band represented one locus. Therefore, the 1470 PCR amplicons represented 1470 distinct loci. Each locus was scored and de-convoluted independently. The 1470 loci (580 SSR primer-derived and 890 STS primer-derived) were anchored onto the BAC-based physical map [13,14], among which, 1020 loci are genetically mapped and 450 loci are unmapped. A proportion of markers (174, 11.6%) could not be de-convoluted because of either false negatives (most cases, 170 markers) or due to apparent amplification of repetitive sequences (14 markers). The 1470 loci (580 SSRs, 890 STSs) mapped to 1184 physical map contigs. The complete dataset used to perform this analysis is provided in additional file 2.

**Figure 2:**
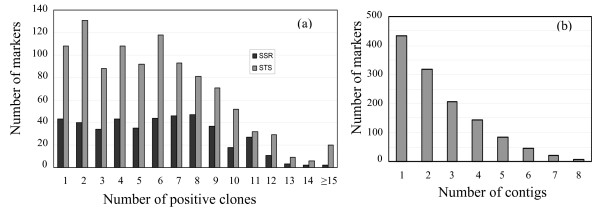
**Distribution of BAC hits and contig/marker**. (a) the distribution of BAC hits/marker using 1470 markers; (b) the distribution of the numbers of identified contigs of each marker.

Figure 2 shows the number of BACs identified per marker. In total, 7,463 clones (1× genome equivalent) were associated with 1470 anchored loci, of which the majority of clones (6,157, 82.5%) were anchored by one marker and 1106 (17.5%) individual clones contained two or more markers. Of 1470 loci, 151 (10.3%) identified a single BAC, 571 (38.8%) identified 2~5 BACs, 607 (41.3%) identified 6~10 BACs and 141 (9.6%) hit more than 10 BACs. A total of 407 (27.9%) markers identified singleton BACs (i.e., not placed in a contig). Therefore, 1053 markers hit clones located in contigs: 349(23.9%) markers hit only one contig, 257 (17.6%) markers (23.4%) identified BACs from two contigs. 447 markers (30.6%) identified BACs from three or more contigs. These results are consistent with the highly duplicated nature of the soybean genome and illustrate the difficulties that the community faces in further defining the soybean physical map. However, the data clearly show that the pooling strategy used is an effective and efficient way to define marker:BAC associations thereby providing anchor points between the soybean genetic and physical maps.

**Figure 3:**
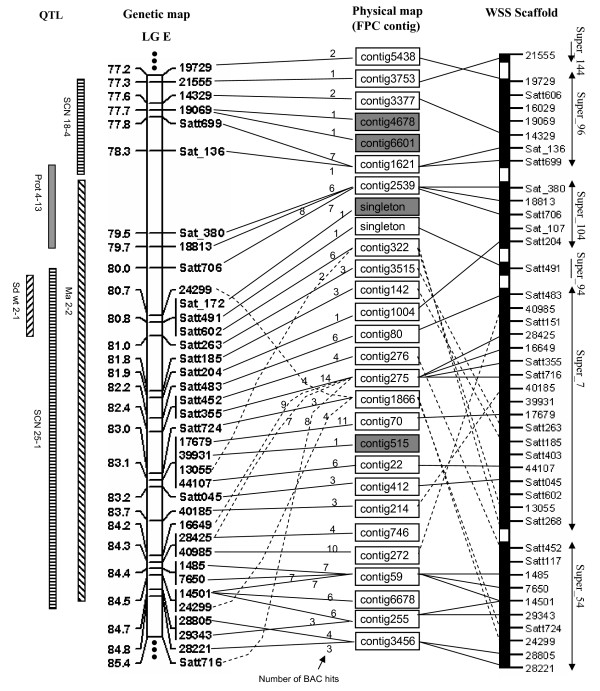
**Example of the integrated map view of a ~8 cM region on LG E**. The genetic map was redrawn based on the integrated genetic linkage map [11]. The QTL name and position refer to the Soybean Breeders Toolbox [34]. The dashed lines indicate the discrepancies of marker alignments between the physical map and genetic map. The highlighted FPC contigs are questionable. The number above the lines connecting genetic markers and contigs is the number of BAC hits. The white bars between highlight bars are gaps between the WSS scaffolds.

An example of integration of the genetic and physical map with 36 genetic markers for a detailed region of ~8 cM length on linkage group (LG) E is illustrated in Figure 3. We combined three sources of data (marker data from the 6-D pools, FPC contigs and their corresponding BESs and 7× assembly (pre-release) of Williams 82 whole-genome shotgun sequences (WSS) provided by Dr. Jeremy Schmutz) to analyze the 8 cM region to determine the degree of colinearity between the three sources of data in order to ascertain the potential difficulties to be encountered in a genome-wide integration. A total of 193 clones hit by 36 markers distributed to 26 FPC contigs ranging from 1 to14 clones/contig with two singletons (Figure 3). Eight of the 26 contigs were targeted by two or more markers. From the alignments between genetic markers and FPC contigs, few discrepancies of marker order between physical and genetic maps are obvious. The position of several markers (Satt716, 24299, 28425, 16649, and 13055) on the genetic map may be not accurate because of genotyping errors. We used sequences of the 36 markers and BESs of the 193 clones to blast against the 7× assembly. The WSS scaffolds were arranged based on the marker order on the genetic map and aligned to FPC contigs if BESs supported the anchoring (Figure 3, Additional file 3). The result indicated that discrepancies of marker order indeed exist between the genetic map and 7× assembly. Marker anchoring by 6-D pool screening was very accurate except for a few contigs with a single hit. Therefore, FPC contigs detected by a single clone tend to be problematic and may be false positives. For the 26 FPC contigs, we estimated the average false positive rate for contig assembling of BAC clones is 14% (Additional File 4).

A detailed analysis of 73 clones identified by 12 SSR markers was conducted to confirm the accuracy of the data obtained from the BAC pool screening. The results revealed an overall false positive rate of 13.9%. However, more than 90% of the false positive clones came from those where only a single clone was detected in a contig. Therefore, to reduce this source of error, we recommend that only overlapping clones detected by a genetic marker or non-overlapped clones detected by multiple genetic markers (assuming no conflict with other nearby markers) should be used as anchoring points for integration of the genetic map and physical map. If we assume the linkage group assignment of the genetic markers used in our study and the assembly of the current FPC contigs all correct, 60% of the contigs with multiple markers would conflict with an anchor marker placed by assignment to a single BAC clone. However, if we remove such markers, then these conflicts drop to only 7%. The real level of conflict may be even lower since we did not eliminate contigs for clearly duplicated regions or those that may be incorrectly assembled. Thus, given the correct parameters, the 6D pool strategy is a powerful way to improve the alignment of the soybean physical and genetic maps.

### *In silico *anchoring of sequence tagged sites

We used the soybean Unigene set (21,700 unigenes) from NCBI [29] as query entries to blast against the non-redundant BAC end sequences (80,700 reads) of the Williams 82 *Bst*Y I library. Using a strict 10^-30 ^e-value cutoff and keeping only one hit with the longest alignment within a blasted BES, we found 9835 unique BES hits for 4152 unigenes with ≥95% sequence identity over regions ≥100 bp in length of the aligned sequences. The hits had an average of 97.6% sequence identity and 287 bp sequence alignment length to the query unigenes. Out of the 4152 unigenes, 2902 (69.9%) had only one hit and 1250 (30.1%) had an average of 5.5 hits (from 2 to 239 hits). Out of the 1250 unigenes that had multiple hits, 293 (7.1%) were located in different contigs (the number of contigs: 2~114). Those unigenes located on few contigs likely represent duplicated regions. Those unigenes hitting several contigs were found to be mostly retroelement-like sequences (additional file 5). A brief summary of the categorized search results is shown in Table 1. The complete blast list is available in the additional file 6. The exact number of new gene-based markers that were unambiguously placed onto the Williams 82 physical map using the in *silico *approach was 2024 (additional file 3). Another 1835 unigenes were located on singleton BACs (1624) or BAC clones (211) without fingerprint data. Most of unigenes located on singleton BACs could be mapped with the aid of manual editing or genetic markers in the same clone.

**Table 1:** Summary of *in silico *mapping with soybean Unigenes

Number of Unigenes	Number of positive clones											
	
	1	2	3	4	5	6	7	8	9–20	20–50	>50	Total
Mapped in one contig	1322	546	131	40	15	4	1	1	7	1	0	2068
Mapped in multiple contigs	80	69	38	15	8	10	5	16	26	23	290	
Mapped in singltons or clones without fingerprinting	1580	150	51	11	1	0	0	0	1	0	0	1794

Total	2902	776	251	89	31	12	11	6	24	27	23	4152

**Figure 4:**
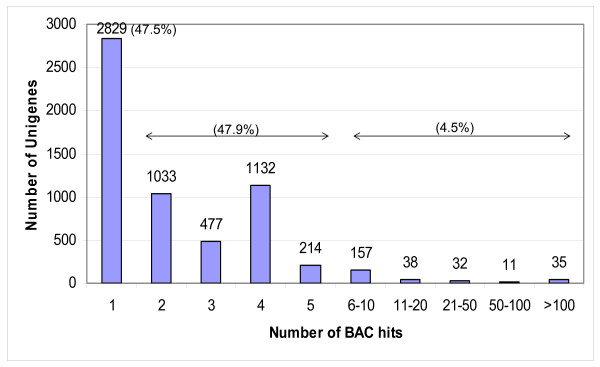
**Distribution of the number of markers according to the number of positive clones for each category of marker**. The soybean Unigene set (21,700 unigenes) served as a query entry to blast against the non-redundant BAC end sequences (80,700 reads) of the Williams 82 *Bst*Y I library. The cutoff parameters were setup as: 10^-30 ^e-value, ≥95% sequence identity and minimum aligned sequence length ≥100 bp.

The *in silico *mapping data showed that the 4152 unigenes were not evenly distributed throughout the 1305 contigs of the most current version of the Williams 82 physical map (Figure 4; [13]). Of these contigs, 458 (35.1%) have only one unigene, 772 (59.1%) contigs have 2~8 unigenes, and 75 (5.7%) contigs have 9~44 unigenes. In addition, among the 1305 contigs anchored by *in silico *mapping of unigenes, 305 contigs were exclusively anchored by *in silico *mapping not by 6-D pool screening. The relative low number of genes used in our analysis prevents firm conclusions. However, it is tempting to speculate that the contigs with multiple unigenes represent gene-rich regions of the genome. However, our data would also suggest that some contigs with higher number of unigenes (e.g., contig1 and contig6136) may be misassembled since the 6-D pooling data revealed marker inconsistencies between the genetic and physical map locations in these contigs. Table 2 shows the difference in the number of BACs anchored for 23 markers for which we obtained results by both PCR (7.43 hits in average) and *in silico *(1.17 hits on average) approaches. The lower number of BAC hits using the *in silico *approach simply reflects the relatively low amount of sequence information available. These results are very similar to the data obtained from analysis of the grapevine physical map [23].

**Table 2:** Comparison of the number of BACs anchored either by PCR screening of the 6-D pools or BLAST searching of the Williams 82 BES

Marker name	Number of BAC anchored by in silico mapping	Number of BAC anchored by PCR screening
AF167556	1	5
AF327903	3	9
AW348668	1	6
AW348889	1	10
AW348942	1	2
AW349052	1	1
AW349078	1	5
AW349154	1	6
AW349256	1	10
AW349806	1	7
AW349953	1	5
AW350435	3	26
AW351080	1	9
BE657482	1	6
BE658220	1	3
BE659529	1	8
BE820710	1	4
BE822259	1	5
BE822505	1	14
BE822673	1	7
D31700	1	7
M64267	1	6
X68702	1	10

Average	1.17	7.43

### Mapping of paralogous sequences

**Figure 5:**
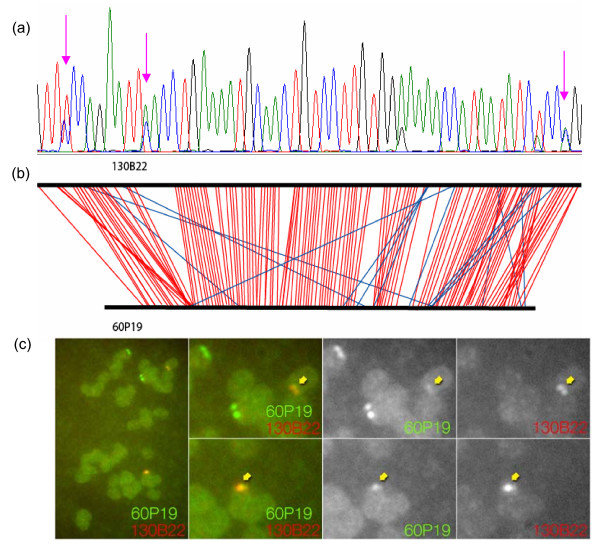
**Anchoring paralogous sequences onto the physical map by 6-D pools and confirmed by FISH**. (a) Paralogous sequences of PCR product. The pink arrow shows nucleotide variations in a sequence read. (b) Alignment between homoeologous BACs. Blue lines show some regions with inconsistent alignment between the two BACs (c) FISH mapping of homoeologous BACs (Gm_WBb0130B22 and Gm_WBb0060P19) anchored by marker 13617. The leftmost panel shows the entire karyotype of a chromosome spread prepared from mitotic root tip cells. The probe for BAC Gm_WBb0130B22 (abbreviated as 130B22) was labeled with green fluorophore and Gm_WBb0060P19 (abbreviated as 60P19) was labeled with red fluorophore; overlap therefore appears as yellow. Right panels are enlarged fields in which the left panels show the merged channels, the middle panels show the green (60P19) channel and the right panels show the red (130B22) channel. The 60P19 probe localizes to two pairs of strong spots (corresponding to the duplicated chromatids of homologous chromosomes) as well as to two pairs of less intense signal that overlap with the 130B22 loci. The yellow arrows indicate overlapping spots.

Primers to amplify paralogs in the soybean genome were identified during the discovery of SNPs via re-sequencing PCR amplicons across six diverse soybean genotypes [11]. Primers were designed from the sequence trace files of PCR products showing 1–5% nucleotide polymorphisms across the 6 genotypes (Figure 5a). A total of 52 STS markers were used to demonstrate the utility of the 6D pool screening method to map paralogous regions. The results of these PCR screens are shown in the physical map [13]. Four of the markers detected only one BAC clone. However, 48 (92.3%) of the markers screened identified multiple clones, with 43 (90%) markers anchored to different contigs. The proportion of BACs anchored to different contigs for these STS markers is much higher than genetically-mapped markers.

Shotgun sequencing of four pairs of BACs anchored by four different STS markers was done to further evaluate the quality of the data. The two randomly-selected clones for each marker were located in different contigs. Each clone was purified before DNA isolation for PCR confirmation. Sequencing of BAC Gm_WBb0080M12 yielded only 54 kb of sequence. However, all of the other BACs gave an average of 150 kb of sequence, representing >1 Mb of combined sequence. A brief summary of the shotgun sequencing results is shown in Table 3. In the alignment fragments, each BAC pair anchored by one marker exhibited a very high level of sequence identity, from 90.4% to 99.2%. This was especially true for BACs Gm_WBb0033D02 and Gm_WBb0027H20, anchored by marker 13757 (GB# AW348725, Apyrase-like protein), which shared 100% identical sequence throughout virtually their entire length. As an example, the alignments of homoeologous sequences between BACs Gm_WBb0130B22 and Gm_WBb0060P19 were illustrated in Figure 5b. We confirmed that the genomic regions corresponding to the two BACs Gm_WBb0130B22 and Gm_WBb0060P19 are duplicated on separate chromosomes using fluorescence *in situ *hybridization (FISH) to soybean mitotic chromosomes (Figure 5c).

**Table 3:** Nucleotide identity between two BACs anchored by the same marker but located at different locations in the physical map

BAC	Sequence length (bp)	Contig	Marker	Matched length (bp)	Aligned length (bp)	Identity (%)
Gm_WBb0100H11	177,735	singleton	14313	47631	53029	90.4
Gm_WBb0057N04	165,162	Contig823	14313			
Gm_WBb0033D02	163,713	Contig1289	13757	158184	158669	99.2
Gm_WBb0027H20	162,558	Contig1173	13757			
Gm_WBb0060P19	113,781	Contig4841	13617	46753	50166	93.3
Gm_WBb0130B22	140,870	singleton	13617			

Gm_WBb0082G12	123,378	Contig690	13879	-	-	-
Gm_WBb0080M12	54,505	singleton	13879			

### Merging small contigs into a big contig

Collectively, the SoyMAP project [30] has placed >7,000 markers onto the Williams 82 physical map via either 6-D pool PCR screening or overgo hybridization. However, approximately 330 floating contigs (lacking any markers) still exist. A total of 240 pairs of primers were designed from these contigs after eliminating repetitive sequences. After primer testing, 163 primer pairs were used for 6-D pool screening in an attempt to merge these floating contigs with larger contigs on the physical map. This same strategy can be used as an efficient way to walk out on contigs spanning large genomic regions of interest. Of those primers tested, 95 (58.3%) were anchored to a clone(s) in the same contig containing the BES used for primer design, while 68 markers were mapped to different contigs (Additional file 2). These results indicate that ~58% of the floating contigs could not be placed onto additional BACs in the *Bst*Y I library pools. The other floating contigs were either extended or merged to larger contigs via the 6-D pool PCR screening.

## Discussion

A 'gold standard' soybean physical map is the stated goal of the soybean community [3] and will be an important resource for the assembly of the whole genome shotgun sequence being generated by the DOE-JGI project. It will be particularly useful to aid assembly of repeat-rich regions such as telomeres, centromeres and duplicated regions that are highly similar at the sequence [8]. The sequence assembly, in turn, can be used to evaluate the accuracy of fingerprint map merges and to identify contig overlaps not recognized by fingerprint comparisons [31]. The degree to which the fingerprint map can be used to guide the assembly of shotgun sequences largely depends on the contiguity and quality of the map. The procedure for fingerprint map construction begins with fingerprint generation, followed by automated fingerprint assembly and manual editing, assisted by contig orientation and merging based on the information of genetic markers anchored on BAC contigs. Manual editing can improve the contiguity by more than an order of magnitude, but the manual editing phase of map construction is time-consuming and requires dedicated and highly trained staff [31]. The integration of physical and genetic maps for a 8-cM region on LG E, assisted with the 7× assembly of whole genome shot gun sequence, demonstrated that the integrated physical map would be more reliable if caution is taken in data clean-up for marker order, questionable clones on a FPC contig or potential errors on the WSS assembly, and a high density genetic map would be helpful for integration of maps.

Although BAC fingerprints are the fundamental data for BAC ordering and contig assembly, the fingerprints have several limitations. For example, it is difficult to assemble large contigs even with several libraries and 15× or more coverage without including bridging clones with minimal overlap [19]. In addition, when analyzing the data at successively higher cut-off values such as >e-20 to examine possible contig mergers, it is difficult to determine contig merges without additional information (e.g., anchored markers). The current soybean physical map represents merges done at e-26; a value chosen since lower stringency merges generated too many Q (questionable) clones [14]. The contig merging errors at a higher cut-off may be caused by genome complexity instead of the repetitive elements [19].

In this study, we analyzed the utility of the 6D pool, PCR approach for efficiently mapping markers to soybean physical map contigs. Specifically, we evaluated 163 BES-based markers for extending and merging floating contigs. Our results showed that 42% of the floating contigs could be merged via overlapping clones identified by the 6-D pool screening. In theory, given additional libraries, this could be an efficient approach for anchoring all of the floating contigs. Collectively, the data show that the 6D pooling strategy provides a powerful approach for improving the soybean physical map by identifying overlapping BAC clones, establishing the relationship between BAC contigs and genetic markers, and merging and extending contigs by placement of BAC singletons. This strategy can also be used efficiently for assembly of locus-specific contigs of long-range, highly duplicated regions (Figure 5) and targeting all members of a specific gene family, such as MIPS [32] and LysM [33]. This approach has a 87.8% success rate in soybean for anchoring genes to BAC clones, which is much higher than 12 × 12 overgo pooled hybridization with ~50% success rate (S. Jackson, Purdue University, personal communication). The use of this BAC pooling strategy for integrated map construction was successfully demonstrated in sorghum [19] and in maize with a much larger genome [20].

The duplicated nature of the soybean genome complicates the integration of the physical and genetic maps. We found that positive clones of a locus-specific marker often mapped to two or more physical map contigs. In addition, it was common to find markers from different regions of the linkage map that mapped to the same contigs, a situation that was previously noted in the assembly of the genotype 'Forrest' physical map [12]. Most of the SSRs and SNP-containing STS may be locus-specific, as defined by single bands on agarose gels, when genomic DNA is amplified. However, single bands were not always found when the BAC pool DNA was used as the PCR template. The pooled BAC DNA was amplified more efficiently due to its lower complexity allowing the detection of paralogous regions. In order to avoid non-specific amplification, the PCR annealing temperature was 52 or 55°C, 5~8°C higher than 47°C that is recommended for most SSR markers [34].

The 6-D pools provide an efficient and cost-effective way to anchor markers onto FPC contigs. One person can screen six markers per day against the 208 pools (49,192 clones) at a cost of about $35 per marker including labor. The method also has the advantage of avoiding radioactive labeling used in hybridization approaches. PCR-based screening of the DNA pools provides anchoring points quickly and unambiguously compared to the traditional hybridization methods since PCR amplicons with different size can be easily distinguished and simultaneously de-convoluted. The PCR products can also be easily isolated and sequenced to provide further confirmation. These features reduce false positives caused by homoeologous sequences from duplicate regions or from the *E. coli *genomic DNA. Therefore, the BAC pools represent a robust, economical method to map genes or new sequences to the integrated map. However, compared to overgo hybridization with pooled probes, the 6-D pool method is not superior in throughput and coverage of BAC clones.

The BAC clones within the 6D pools represent about 6.6 genome equivalents and, therefore, it was expected that approximately 6~7 positive BAC clones would be identified per marker. However, the average number of BAC clones per marker was 5.8, which was lower than expected. Several reasons were thought to cause this [20]: 1) failure to amplify the amplicons in one or more dimensions of the 6 pools since deconvolution requires amplification in all six dimensions, 2) failure to identify all clones of highly represented sequences due to occlusion, 3) no representation of the region the marker was designed from in the BstY I BAC library, 4) absence of the locus within the subset of the BAC library used to construct the BAC pools, or 5) over estimation of the average insert size of BAC clones.

We found that marker order between the genetic and physical maps was not always consistent (Figure 3). Several reasons may explain this; such as, some genetic markers may not be accurately mapped due to the small size of mapping populations used [10,11], genotyping errors, or skewing of marker segregation; the clones containing the genetic markers may be misplaced on the FPC contig based physical map; or genome duplication may exist within a linkage group (such as contig59, contig6678 and contig255 anchored by STS marker 14501 in Figure 3). To clarify these issues, BES-based markers should be used to screen all overlapped BAC clones containing a given locus. If the target clone is placed correctly, nearby loci tend to be present in many of the same overlapped clones (Additional file 4).

The *in silico *anchoring approach was used to place markers onto FPC contigs. We screened the entire set of unigenes (21,700) against the BES from the *Bst*Y I library. The 80,700 BAC-end sequences added up to a total of about 56.5 Mb of sequence, which roughly corresponds to 0.05 genome equivalents. *In silico *mapping provides a quick means to estimate gene-rich contigs and also to position the gene onto a BAC, which provides a physical map location and, by association, a genetic map location.

## Conclusion

We demonstrated that the six-dimensional soybean BAC pools can be efficiently used to anchor markers to soybean BACs despite the complexity of the soybean genome. In addition to anchoring genetic markers onto the physical map, the pools can be used to target duplicated regions that could confound the contig assembly, merge small contigs and rapidly map members of a gene family. In addition, the *in silico *mapping approach was also useful to quickly obtain additional marker:BAC associations in soybean that could benefit physical and genetic map integration and target genes of interest.

## Methods

### BAC library construction

The GMW2 (Gm_WBb) library was constructed using the *Bst*Y I site of pCUGIBAC1 (Chris Saski, Clemson University Genomics Institute), and has 92,000 clones with an average insert size of 150 Kb representing 12× haploid genome equivalents.

### BAC pooling Strategy

#### Stack design and layout

The pooling strategy used in the present study followed that used for sorghum [19] with some modifications necessary due to the soybean genome size. The pooling scheme is illustrated in Figure 1, 128 384-well microtiter plates were arranged into a cubic stack which consisted of 32 layers with each layer containing four 384-well plates. The four plates in a layer were arranged in a 2 × 2 format, which resulted in each layer containing wells arranged in 32 rows × 48 columns. A total of 49,152 BAC clones were pooled in six distinct directions to generate 208 unique pools representing ~6.6 fold genome equivalent. The six dimensional pools were named as Plate Pool (PP), Face Pool (FP), Side Pool (SP), Row Pool (RP), Column Pool (CP) and Diagonal Pool (DP), respectively. Five of the six pool types (PP. FP, RP, CP and DP) were composed of 32 pools each containing 1536 BACs. The SP was composed of 48 pools each containing 1024 BACs. Thus, every BAC of the 49,152 BACs was sampled only once in any pool type.

#### Pooling Strategy

BAC pools were generated by pooling the BAC clone culture in the conceptually-stacked microtiter plates in six different matrices. Each pool represents the intersection of a plane within the cube (Figure 1). Plate pool (PP) was produced from the four plates of each layer. Face pool (FP), which was defined as a plane parallel to the front face surface of the stack, consisting of BACs that share the same *y*-coordinate. Side pool (SP), which was defined as a plane parallel to the surfaces left and right of the stack, consisted of BACs that share the same *x*-coordinate. Row pools (RP) were established as follows: pool all BACs together in Row R (*y*) in Layer P(*z*) with the same number y + z to form the RP (y+z) (i.e. all of the BACs from layer 1 row 2 are combined with those from layer 2, row 1 to form RP3 and so on for other combinations). When the R + P value is greater than 32, then 32 was subtracted from R + P to give the correct row pool, i.e., BACs in row 20 on layer 20 were pooled into RP8 (20+20 = 40-32 = 8). Column pools (CP) were established in the same manner as row pools by column C (*x*) added to layer P (*z*). CP equals column number plus layer number CP = C + P, when the C + P value is greater than 32 but smaller than 2 × 32, then 32 was subtracted from C + P to form the given column pool, i.e., BACs in column 20 of layer 20 were pooled into CP8 (20+20 = 40-32 = 8); when the C + P value is greater than 2 × 32, then 64 was subtracted from C + P to form the correct column pool, i.e., BACs in column 45 of layer 20 were pooled into CP1 (45+20 = 65-64 = 1). All wells from row R (*y*) and column C (*x*) consisted of diagonal pools (DP). The pooling method of DP was similar with the one used in CP. finally, the six types of BAC pools resulted in 208 pools. In order to test primers and optimize PCR efficiently, we generated a mixed pool that contained all BACs from the first set of culture after inoculation.

### BAC DNA isolation from pools

Each time one of the six pool types was prepared, the 128 384-well microtiter plates comprising the pooling stack were inoculated with BAC stocks. BACs were inoculated from a frozen stock plate using a 384-well pin tool (Matrix, CA) into microtiter plates containing 70 μl LB media plus 12.5 μg/ml chloramphenicol per well and the plates were incubated overnight at 37°C. The next day, another set of 128 384-well microtiter plates with TB medium plus 12.5 μg/ml chloramphenicol were inoculated with the BACs from the prior night's LB plates and grown overnight using the same procedure as the day before. The third day the plate positions were assigned and labeled in series numbers located in the conceptual stack. For subsequent BAC pooling, the plate inoculated from the same stock plate would use the same assigned number. For BAC pooling, 45 μl of culture was removed from each well using 8- or 12-channel multi-pipette for five (plate, face, side, row, and column) matrices and 8-channel adjustable pipette (Matrix, CA) for the diagonal matrix. The BAC cultures were placed in sterile containers with each container defining a given pool. BAC DNA isolation was performed using Qiagen BAC DNA Isolation Kit (Qiagen Inc.).

### Choice of markers for anchoring and PCR screening

All SSR markers were chosen from the soybean composite map [10], the marker information can be gained from the Soybean Breeders Toolbox [34]. The STS primers were chosen from those containing SNP markers among different genetic mapping populations and that were genetically mapped [11].

PCR was conducted in a 15 μl reaction containing BAC DNA 10 ng, 1 × PCR buffer, 2.5 mM MgCl_2_, 200 μM dNTP, 2 pmol of each primer, 1% PVP, 1.0 U Taq Polymerase (GenScript Corp.). The samples were preheated at 95°C for 3 min, subjected to 35 PCR cycles of 94°C for 30 s, 58°C for 30 s and 72°C for 1 min, and then a final extension step of 72°C for 7 min. PCR products were separated using 3%~3.5% 1× TBE agarose gel electrophoresis. Each sample on agroase gel was labeled with its corresponding pool name to avoid any errors when scoring. The PCR amplicon was scored in binary format, i.e. present or absent.

### FISH of soybean homoeologous BACs

Fluorescent in situ hybridization using homoeologous BACs was carried out as previously described by Zhang et al. [33], except that Qiagen Large Construct Kit (Qiagen Inc.) purified BAC DNA was used for nick-translated probes.

### De-convolution of pool data

Based on the Perl script initially used in sorghum by Klein et al. [19], we made some modifications to reflect the layout of the 6-D pools. The program allows the candidate BAC clones to be de-convoluted from the marker data obtained using the BAC DNA pools.

In this system, any well in a plate can be located by any three pool types. So only three degrees of freedom (*x*, *y*, *z*) are present in the system even though there are six pool types. The procedure of data de-convolution was as follows: (1) three predictions – using the presence of a PCR amplicon in plate pools and face pools to predict the presence of row pools, plate pools and side pools to predict column pools, and face pools and side pools to predict diagonal pools; (2) merging three predictions through common pool type into combinations of six pool types, each combination would correspond to an individual clone as a candidate positive; (3) identifying unique pools in any pool types from the list of candidates, i.e. a positive clone would be called only when a PCR amplicon exists in all six pool types and at least one pool type is unique. This three step de-convolution was used for data analysis in sorghum [19] and maize [20]. The multiple predictions from different pool types actually are a confirmation process that eliminates many alternative addresses and detects a marker multiple times in the appropriate pools before it is accepted as positive. Meanwhile, we noticed that this approach gives a high level of false negatives although it greatly reduces the frequency of false-positives. Based on the preliminary assembly of FPC contigs, we also identified positive clones that were located in the same contig even though, in some cases, they were not uniquely detected. The de-convolution of 6-D pool PCR screening data in this way provides a high level of confidence for marker anchoring to overlapped clones.

## Authors' contributions

XLW participated in the design of the experiment, led the pool construction, carried out data deconvolution, and drafted the manuscript. GHZ carried out PCR assays. SDF carried out FISH assay. PC characterized genetic markers. GS and HTN participated in the design of the experiment, contributed to data interpretation and revision of the manuscript. All authors have read and approved the final version of the manuscript.

## Supplementary Material

Additional file 1 PCR screening workflow. The workflow for PCR screening of 6-D pools is illustrated. Click here for file

Additional file 2 The complete list of mapped markers using 6D pools. The data provided included all information for the 1541 markers about marker name, primer sequence, linkage group, BAC clones targeted and FPC contig number. Click here for file

Additional file 3 Placement of markers and BES of the 8 cM-region on LG E. The dataset shows the placements of 36 markers and BES of 193 clones located in the 8 cM-region on LG E onto the 7× soybean assembly. Click here for file

Additional file 4 Summary of BES anchoring to 7× assembly. The data is summarized to show the percentage of BAC clones anchored to the 7× assembly based on the placements of markers and BES in additional file 3. Click here for file

Additional file 5 Annotation of unigenes mapped by *in silico *approach. The data provided the information about annotation of unigenes mapped by *in silico *approach and the number of BAC hits. Click here for file

Additional file 6 The complete blast list of *in silico *mapping. The dataset shows the results of blasting soybean unigenes against BES of *Bst*Y I BAC library. Click here for file
