# Supplementary figures and images for: Genetic marker anchoring by six-dimensional pools for development of a soybean physical map
Source: BMC Genomics. 2008 Jan 22;9:28. doi: 10.1186/1471-2164-9-28 (PMC2259328; doi:10.1186/1471-2164-9-28)

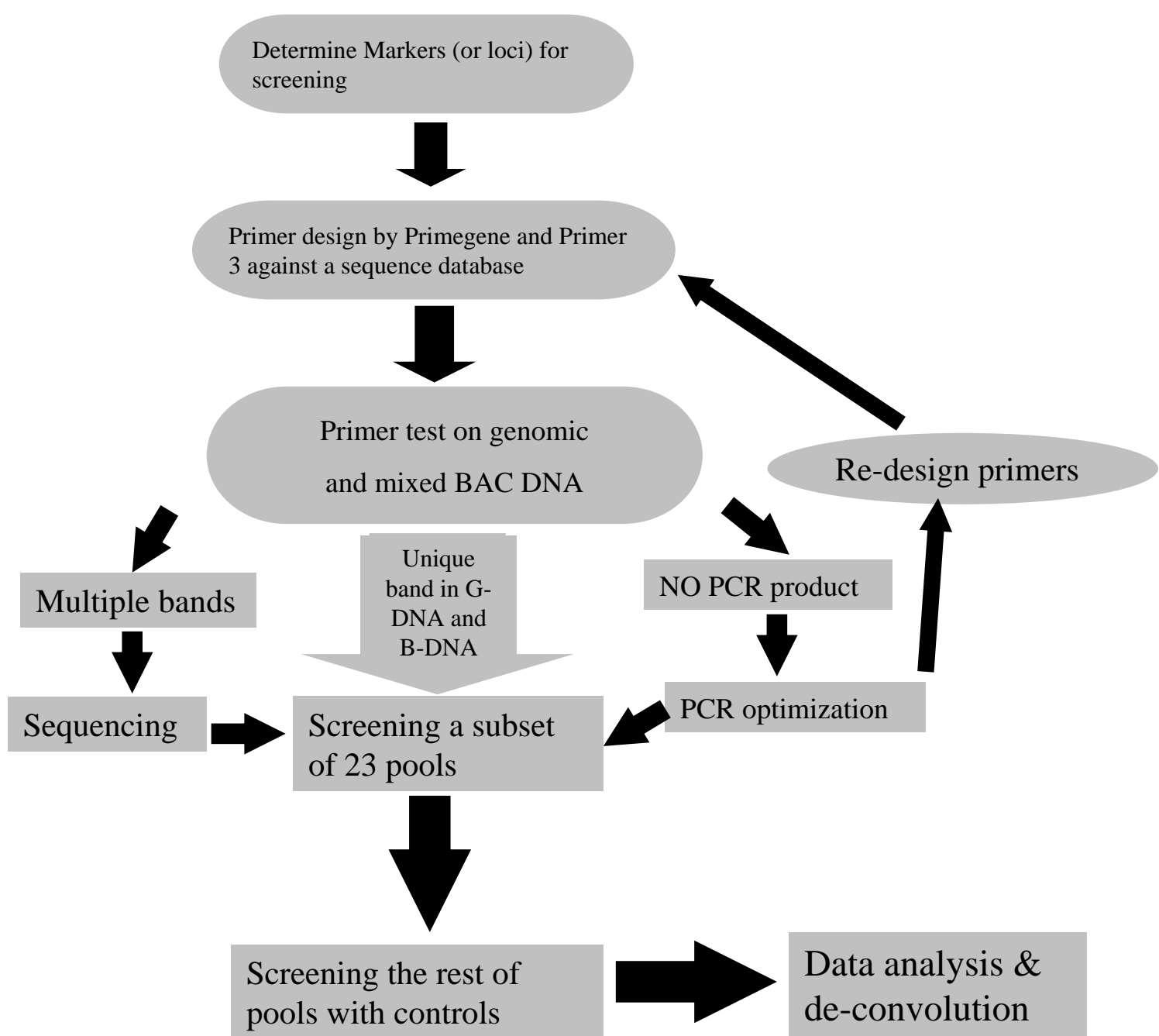

Supplement: Additional file 1 — PCR screening workflow. The workflow for PCR screening of 6-D pools is illustrated. [file 1471-2164-9-28-S1.pdf]
